# 

**Published:** 1912-03

**Authors:** 


### THE BRISTOL

### Medico-Chirurgical Journal.

A JOURNAL OF THE MEDICAL SCIENCES FOR THE
WEST OF ENGLAND AND SOUTH WALES

*PUBLISHED UNDER THE AUSPICES OF*

*THE BRISTOL MEDICO-CHIRURGICAL SOCIETY.*

EDITOR:
R. SHINGLETON SMITH, M.D., B.Sc.,

WITH WHOM ARE ASSOCIATED
J. MICHELL CLARKE, M.A., M.D., J. LACY FIRTH, M.S., M.D.,
AND JAMES SWAIN, M.S., M.D.

ASSISTANT-EDITOR:
P. WATSON WILLIAMS, M.D.

EDITORIAL SECRETARY:
J. A. NIXON, B.A., M.B.

"Scire est nescire, nisi id me
Scire alius sciret."

VOL. XXX.

BRISTOL: J. W. ARROWSMITH LTD.
LONDON: J. & A. CHURCHILL, 7 GREAT MARLBOROUGH STREET.
1912.

R31
.B8

J.W. Arrowsmith Ltd. Publishers Bristol

### CONTENTS OF VOLUME XXX.

Page

Lister: An Appreciation by ROBERT ROXBURGH, M.B., F.R.C.S. Ed. .. 1

Direct Laryngoscopy, Bronchoscopy and Œsophagoscopy. By P. WATSON-WILLIAMS, M.D. Lond. .. .. .. .. .. 11

A Case of Chronic Interstitial Pancreatitis of Uncertain Origin. By J. MICHELL CLARKE, M.A., M.D. Cantab., F.R.C.P. .. .. 25

Operations on the Uterus and Appendages during Pregnancy. By W. C. SWAYNE, M.D. Lond. .. .. .. .. .. .. 31

Some Cases illustrating the Difficulty and Importance of Early Diagnosis in Acute Intestinal Obstruction. By ERNEST W. HEY GROVES, M.S. Lond., F.R.C.S... .. .. .. .. 37

Some Observations on Tuberculosis of the Nose and Pharynx. By A. J. WRIGHT, M.B. Lond., F.R.C.S. .. .. .. .. 45

Some Gall-Bladder Cases presenting Features of Interest. By E. H. E. STACK, M.B. B.C. Cantab., F.R.C.S. .. .. .. 49

The End-Results of Forty-one Operations for Internal Derangements of the Knee-Joint. By A. RENDLE SHORT, M.D., B.S., B.Sc. Lond., F.R.C.S. .. .. .. .. .. .. .. 52

Intra-Cranial Complications of Ear Disease: a Discussion at the Bristol Medico-Chirurgical Society, March 13th, 1912. Introduced by J. MICHELL CLARKE, M.A., M.D. Cantab., F.R.C.P., and J. LACY FIRTH, M.S. Lond., F.R.C.S. .. .. .. .. 97

The Differential Diagnosis of Swelling in the Breast. By CHARLES A. MORTON, F.R.C.S. .. .. .. .. .. .. .. 121

An Enemy of the People—Tuberculosis and Natural Selection. By D. S. DAVIES, M.D. Lond. .. .. .. .. .. .. 135

The Artificial Production of Pneumothorax in Phthisis by Injection of Nitrogen. By HUBERT CHITTY, M.B., M.S. Lond., F.R.C.S. 141

Notes on an Unusual Case of Hodgkin's Disease. By E. LEONARD LEES, M.D. Edin., and F. H. EDGEWORTH, M.D. Cantab., D.Sc. Lond. .. .. .. .. .. .. .. .. .. 150

3/25/17

CONTENTS.

| | Page |
|---|---|
| A Case of the Heroin Habit. By J. ODERY SYMES, M.D. Lond. | 153 |
| Notes on "Some Dreams and their Significance." By Sir GEORGE H. SAVAGE, M.D. Lond., F.R.C.P. | 156 |
| Tuberculosis. By ROBERT ROXBURGH, M.B. Edin. | 193 |
| Some Remarks on Insanity; its Classification and Nomenclature. By F. St. JOHN BULLEN, M.R.C.S. Eng. | 205 |
| The Right and the Wrong Side of the X-Ray Picture; Has a Mistake been Made? By W. COTTON, M.A., M.D. Edin. | 227 |
| The Relationship between Toxicity and Chemical Reactivity in Certain Benzene Derivatives. By OLIVER C. M. DAVIS, D.Sc. | 237 |
| Raynaud's Disease. By DAVID A. ALEXANDER, M.B., Ch.B. Vict. | 247 |
| Robert Fletcher. By Sir WILLIAM OSLER, Bart., M.D., F.R.S. | 289 |
| On some Diseases Bearing Names of Saints. By ROBERT FLETCHER, M.D. Columbia and Bristol, M.R.C.S. | 295 |
| The Problem of Medical Education. By WALTER C. SWAYNE, M.D. Lond. and Bristol. | 316 |
| The Barber Surgeons. By GEORGE PARKER, M.D., M.A. Cantab. | 327 |
| Tuberculin and Immunity. By JOHN WM. TAYLOR, M.D. Bristol | 338 |
| The Bristol Medical Library. By C. KING RUDGE, M.R.C.S. Eng., L.R.C.P. Lond. | 344 |
| PROGRESS OF THE MEDICAL SCIENCES | 64, 251, 350 |
| REVIEWS OF BOOKS | 74, 160, 262, 359 |
| EDITORIAL NOTES | 84, 174, 274, 367 |
| NOTES ON PREPARATIONS FOR THE SICK | 84, 181, 276, 369 |
| THE LIBRARY OF THE BRISTOL MEDICO-CHIRURGICAL SOCIETY | 87, 184, 280, 373 |
| MEETINGS OF SOCIETIES | 89, 187, 282, 376 |
| OBITUARY | 93, 285, 382 |
| LOCAL MEDICAL NOTES | 94, 191, 287, 383 |

### The Library of the Bristol Medico-Chirurgical Society.

---

*The following donations have been received since the publication of the List in December:*

*February 29th,* 1912.

| | |
|---|---|
| L. M. Griffiths .. .. .. .. .. | 9 volumes. |
| J. A. Nixon, M.B. (1) .. .. .. .. | 1 volume. |

Mr. L. M. Griffiths has presented one framed picture, and Mr. James Taylor an unframed portrait.

### EIGHTY-THIRD LIST OF BOOKS.

The titles of books mentioned in previous lists are not repeated.

The figures in brackets refer to the figures after the names of the donors, and show by whom the volumes were presented. The books to which no such figures are attached have either been bought from the Library Fund or received through the *Journal.*

**Batten, F. E.** .. .. *Poliomyelitis in Relation to the Spread of Infection by Schools* .. .. .. .. .. 1911

**Berkart, J. B.** .. *On Bronchial Asthma* .. Abridged 3rd Ed. 1911

*British Pharmaceutical Codex, The* .. .. .. .. .. .. .. .. 1911

**Burghard, Sir W. W. Cheyne and F. F.** *A Manual of Surgical Treatment.* New Edition. (Revised by T. P. Legg and A. Edmunds) .. Vol. I 1912

**Cheyne and F. F. Burghard, Sir W. W.** *A Manual of Surgical Treatment.* New Edition. (Revised by T. P. Legg and A. Edmunds) .. Vol. I 1912

**Cheyne, C. C.** [Ed.] *A System of Surgery* .. .. .. Vol. I 1912

**Davidson, F.** .. *Sight Testing for the General Practitioner* 5th Ed. 1912

**Denslow, L. N.** .. *A Surgical Treatment of Locomotor Ataxia* .. 1912

**Dickey, J. S.** .. *Applied Anatomy of the Lungs and Pleural Membranes* .. .. .. .. .. .. .. 1911

**French, H.** .. .. *Medical Laboratory Methods and Tests* 3rd Ed. 1912

**Gauchier, E.** .. *Diseases of the Skin* (Tr. and Ed. by C. F. Marshall) .. .. .. .. .. .. .. .. 1910

**Griffiths, F. G.** .. *Studies in Pulmonary Tuberculosis* .. .. 1911

**Hill, W.**.. .. .. *On Gastroscopy* .. .. .. .. .. .. .. .. .. 1912

**Hurst, A. M. Marshall and C. H.** *Practical Zoology* (Revised by F. W. Gamble) .. .. .. .. 7th Ed. 1912

**Jee, Sir B. S.**.. .. *A Short History of Aryan Medical Science* .. 1896
**Lewis, T.** .. .. *The Mechanism of the Heart Beat* .. .. 1911
**Macewen, Sir W.**.. *The Growth of Bone*.. .. .. .. .. .. .. 1912
**Marie, P.** [Ed.] .. *La Pratique Neurologique* .. .. .. .. 1911
**Marshall, G. B.** .. *A Manual of Midwifery* .. .. .. .. 1911
**Marshall and C. H. Hurst, A. M.** *Practical Zoology* (Revised by F. W. Gamble) .. .. .. .. 7th Ed. 1912
**Parsons and W. Wright, F. G.** *Practical Anatomy*.. .. 2 Vols. 1912
**Pickerill, H. P.** .. *The Prevention of Dental Caries and Oral Sepsis* .. .. .. .. .. .. .. .. .. 1912
**Schönwerth, A.** .. *Chirurgisches Vademekum* .. .. .. .. 1912
**Stelwagon, H. W.** *Diseases of the Skin* .. .. .. 6th Ed. 1911
**Stothard, R. T.** .. *Psychoneurology* .. .. .. .. .. (1) 1870
**Tubby, A. H.** .. *Deformities, including Diseases of Bones and Joints* 2 Vols. .. .. .. .. 2nd Ed. 1912
**Tunstall, F. J. Warwick and A. C.** *First Aid* .. .. .. 7th Ed. 1911
**Warwick and A. C. Tunstall, F. J.** *First Aid* .. .. .. 7th Ed. 1911
**Wright, F. G. Parsons and W.** *Practical Anatomy*.. .. 2 Vols. 1912

### TRANSACTIONS, REPORTS, JOURNALS, &c.

American Association of Obstetricians and Gynecologists, Transactions of the .. .. .. .. .. .. .. .... .. Vol. XXIII 1910

American Journal of Obstetrics, The .. .. .. .. Vol. LXIII 1911

American Ophthalmological Society, Transactions of the Vol. XII, Pt. 3 1911

Boston Medical and Surgical Journal, The.. .. Vol. CLXIV 1911

Bristol Directory, Wright's .. .. .. .. .. .. .. .. .. .. .. 1912

British Medical Journal, The .. .. .. .. .. Vol. II for 1911

Clinical Journal, The .. .. .. Vols. XXXVII, XXXVIII 1910–11

Deutsche Zeitschrift für Chirurgie .. .. .. Bds. CVIII, CIX 1911

English Catalogue of Books, The, for 1910 .. .. .. .. .. .. 1911

Hazell's Annual .. .. .. .. .. .. .. .. .. .. .. .. .. 1912

Journal of Medical Research, The .. .. .. .. .. Vol. XXIV 1911

Journal of Obstetrics and Gynæcology of the British Empire Vol. XIX 1911

Journal of the American Medical Association, The .. Vol. LVI 1911

Lancet, The .. .. .. .. .. .. .. .. .. .. .. Vol. II for 1911

Medical Directory, The .. .. .. .. .. .. .. .. .. .. .. 1912

Medical Record, The .. .. .. .. .. .. Vol. LXXIX 1911

Minerva .. .. .. .. .. .. .. .. .. .. .. .. .. .. .. 1911–12

Münchener medizinische Wochenschrift .. .. .. Bd. I for 1911

Nordiskt Medicinskt Arkiv .... .. .. .. .. .. .. .. .. 1909

Prescriber, The .. .. .. .. .. .. .. .. .. .. .. Vol. V 1911

Press Guide, Willing's .. .. .. .. .. .. .. .. .. .. .. 1912

Proceedings of the Royal Society of Medicine .. .. .. Vol. IV 1911

Progressive Medicine .. .. .. .. .. .. .. .. .. Vol. III 1911

Quarterly Journal of Medicine, The .. .. .. .. Vol. IV 1910–11

Revue de Gynécologie .. .. .. .. .. .. .. .. .. Tom. XV 1910
Wellcome Tropical Research Laboratories, Fourth Report of the Vol. A—Medical 1911
Whitaker's Almanack .. .. .. .. .. .. .. .. .. .. .. .. .. .. 1912
Year-Book of Scientific and Learned Societies .. .. .. .. .. .. .. 1911

### MEETINGS OF SOCIETIES.

### Bristol Medico-Chirurgical Society.

### Local Medical Notes.

**Markham Skerritt Memorial.**—A few years ago it was decided to endow the Chair of Anatomy as a memorial to the late Dr. Markham Skerritt, who was for many years Dean of the Medical Faculty and Professor of Medicine; but sufficient funds for this purpose did not come in, and it has been decided that the sum then collected (£337) should be handed to the University authorities for the purpose of establishing a biennial prize for original work in medical science.

**Association of Alumni.**—The Committee of this Association recently held in the Council Chamber of the University an Exhibition of items of Bristol interest, literary and scientific, including amongst others the works, portraits, etc., of John Addington Symonds, J. C. Prichard, Bishop Hall, Mary Carpenter, and a very interesting collection of MSS. which had been discovered in the Bristol Cathedral. During the evening a short lecture was given on each section of the exhibition, and greatly added to the enjoyment of the evening.

**The National Association for the Prevention of Consumption** is actively engaged in forming a library and bureau for the collection of all matters relating to pulmonary tuberculosis from every point of view and in all countries. It is intended that such information shall be available not only to members of the medical profession, but to the public at large. Valuable assistance would be rendered if Medical Officers of Health, School Medical Officers and Medical Superintendents or Secretaries of Hospitals, Sanatoria, Tuberculosis Dispensaries and Open-air Schools would kindly place the Association on their distribution list in respect of Annual Reports or other documents bearing on the question of consumption. Books, pamphlets and reprints of articles from physicians and social workers in general would also be gladly received at 20 Hanover Square, London.

**A.P.M.: Ninth Congress.**—The Ninth Annual Congress of the Association Internationale de Perfectionnement Scientifique (A.P.M.), under the high patronage of the French Government, will take place from the 3rd to the 31st August, 1912, in the Balkans, in Turkey and in Greece.

The Congress will be opened in Evian-les-Bains or Thonon-les-Bains (Lac de Genève), and will be continued in the following order: Venise (via Simplon), Trieste, Grottes d'Adelsberg, Agram, Belgrad, Descente du Danube, Passes de Kazan, Portes de Fer, Bucarest, Sofia, Constantinople, Mytilène, Smyrne, Athènes, Phalère, Eleusis, Corynthe, Olympia, Corfou, Bologne (via Brindisi). The last meeting will be held at Aix-les-Bains.

The programme of the Congress was published in the January number of the *Review* of this Association. A copy of this special number may be seen in the Bristol Medical Library.

For further particulars write enclosing international reply stamp 2½d. to the President, Head Office, A.P.M., 12 Rue François-Millet, Paris XVI.

Dr. Ghislain Houzel, *General Secretary.*

### EXAMINATION SUCCESSES.

UNIVERSITY OF BRISTOL.—*M.B., Ch.B.:* E. V. Foss, J. R. Kay-Mouat, P. Moxey. *Part I:* G. F. Fawn, W. P. Taylor.

*B.D.S.:* G. F. Fawn.

*D.P.H.:* Thos. J. Williams.

UNIVERSITY OF LONDON.—*M.B., B.S.:* R. M. Wright, W. W. Pratt. *Part I:* H. B. Parker.

*First Examination for Medical Degrees:* Arthur H. Morris.

CONJOINT BOARD.—*M.R.C.S., L.R.C.P. Lond.:* T. E. Ashley, J. W. Whiteman. *Biology:* J. H. C. Eglinton. *Anatomy and Physiology:* C. N. Vaisey, J. P. Adcock. *Surgery:* L. C. Watkins-Baker.*

L.D.S.—*Chemistry:* Marjorie J. White, A. H. Virgin. *Physics:* Marjorie J. White.

I.M.S.—P. J. Veale, M.B., B.S. Lond. (2nd place).

R.A.M.C.—W. W. Pratt, M.B., B.S. Lond.) 5th place).

* Completes examination.

### APPOINTMENTS.

**University of Bristol.**—E. W. Hey Groves, M.S., F.R.C.S., has been appointed Lecturer on Surgery to Dental students.

**Bristol Royal Infirmary.**—The following have been appointed :—A. E. V. Spill, L.D.S. Eng., Dental House Surgeon; C. H. Hart, House Physician; R. C. Clarke, M.B., Ch.B. Bristol, House Surgeon; N. C. Scott, M.B., House Surgeon, Throat and Nose Department; R. S. S. Statham, M.B., Ch.B. Bristol, Obstetric House Surgeon.

**Bristol General Hospital.**—A. C. S. Cottam, L.D.S. Eng., has been appointed Dental House Surgeon.

### Bristol Medico-Chirurgical Society.

### President.
C. A. MORTON, F.R.C.S.

### President-Elect.
WALTER C. SWAYNE, M.D., B.S. Lond., Bristol.

### Committee.

*Ex-officio*

- B. M. H. ROGERS, B.A., M.D., B.Ch. Oxon. (Retiring President.)

- R. SHINGLETON SMITH, M.D. Lond. (Editor of *Journal*).

- C. K. RUDGE, M.R.C.S. (Hon. Librarian).

- J. A. NIXON, B.A., M.B. Cantab. (Editorial Secretary).

*Elected.*

G. MUNRO SMITH, M.R.C.S. } Ex-Presidents.
J. MICHELL CLARKE, M.A., M.D. Cantab. } Ex-Presidents.
P. WATSON-WILLIAMS, M.D. Lond.
G. PARKER, M.A., M.D. Cantab.
I. WALKER HALL, M.D. Vict.
F. LACE, F.R.C.S.
J. LACY FIRTH, M.D., M.S. Lond.
JOHN WALLACE, M.B., C.M., B.Sc. Edin.

### Honorary Secretary.

J. A. NIXON, B.A., M.B. Cantab.

Medical Practitioners wishing to join the Society are requested to communicate with the Hon. Secretary, Dr. NIXON, 19 Mortimer Road, Clifton, Bristol. The Annual Subscription is 21/-.

---

*Extracts from Journal By-laws.*

4.—The *Journal* shall be delivered free to Subscribers and also to Members of the Society whose subscriptions are not in arrear.

6.—The Annual Subscription to the *Journal* for those who pay before the 1st of July shall be 5/-, post free, or 6/- if paid after that date.

---

Communications intended for insertion in the *Journal* should be sent to the Editor, Dr. SHINGLETON SMITH, Deepholm, Clifton Park, Clifton, Bristol.

Books for Review and Exchange Journals should be sent to the Assistant-Editor, *Bristol Medico-Chirurgical Journal*, Medical Library, The University, Bristol.

Communications referring to the delivery of the *Journal* should be sent to the Editorial Secretary, Dr. J. A. NIXON, 19 Mortimer Road, Clifton, Bristol, to whom Subscriptions are to be paid *in advance*.

Cases for Binding Vols. I-XXIX are now ready, and may be obtained, price *7d.* each (postage *2d.* extra), upon application to J. W. ARROWSMITH Ltd., Quay Street, Bristol ; or the *Journal* will be bound, including Case, for 1/3 per volume.

### Bristol Medico-Chirurgical Society.

### PAST PRESIDENTS.

1874—75 FREDERICK BRITTAN, M.D., B.A. Dub.
1875—76. ROBERT WILLIAM COE.
1876—77. SAMUEL MARTYN, M.D. Ed.
1877—78. AUGUSTIN PRICHARD, M.D. Berlin.
1878—79. HENRY EDWARD FRIPP, M.D. Würzburg.
1879—80. WILLIAM MICHELL CLARKE.
1880—81. JOSEPH GRIFFITHS SWAYNE, M.D. Lond.
1881—82. EDWARD LONG FOX, M.D. Oxon.
1882—83. JAMES GEORGE DAVEY, M.D. St. And.
1883—84. WILLIAM JOHNSTONE FYFFE, M.D., B.A. Dub.
1884—85. GEORGE FORSTER BURDER, M.D. Aberd.
1885—86. WILLIAM HENRY SPENCER, M.D., M.A. Cantab.
1886—87. FRANCIS POOLE LANSDOWN.
1887—88. LEMUEL MATTHEWS GRIFFITHS.
1888—89. ROBERT SHINGLETON SMITH, M.D., B.Sc. Lond.
1889—90. NELSON CONGREVE DOBSON.
1890—91. SAMUEL HENRY SWAYNE.
1891—92. FRANCIS RICHARDSON CROSS, M.B. Lond.
1892—93. EDWARD MARKHAM SKERRITT, M.D., B.S., B.A. Lond.
1893—94. JAMES GREIG SMITH, M.B., C.M., M.A. Aberd., F.R.S.E.
1894—95. ALFRED JAMES HARRISON, M.B. Lond.
1895—96. ARTHUR WILLIAM PRICHARD.
1896—97. ALFRED EDWARD AUST LAWRENCE, M.D., C.M. Aberd.
1897—98. JOHN EDWARD SHAW, M.B., C.M. Ed.
1898—99. ROBERT ROXBURGH, M.B. Ed.
1899—00. WILLIAM HENRY HARSANT.
1900—01. DAVID SAMUEL DAVIES, M.D. Lond.
1901—02. BARCLAY JOSIAH BARON, M.B., C.M. Ed.
1902—03. GEORGE MUNRO SMITH.
1903—04. JAMES PAUL BUSH, C.M.G.
1904—05. REGINALD EAGER, M.D. Lond.
1905—06. JOHN DACRE.
1906—07. JAMES TAYLOR.
1907—08. HENRY WALDO, M.D., C.M. Aberd.
1908—09. JOHN MICHELL CLARKE, M.D. Cantab.
1909—10. JAMES SWAIN, M.D., M.S. Lond.
1910—11. BERTRAM MITFORD HERON ROGERS, B.A., M.D., B.Ch. Oxon.

1912.

### LIST OF SUBSCRIBERS

TO THE

### Bristol Medico-Chirurgical Journal.

### HONORARY MEMBERS OF THE SOCIETY.

| | |
|---|---|
| Owen, Sir Isambard, D.C.L., M.D. | Hereford House, Clifton. |
| Dobson, N. C., F.R.C.S. ... ... ... | 16 College Road, Clifton. |
| Griffiths, L. M., M.R.C.S. ... ... | 11 Pembroke Road, Clifton. |
| Smith, R. Shingleton, M.D., B.Sc., F.R.C.P. ... ... ... ... ... ... | Deepholm, Clifton Park. |

### SUBSCRIBERS.

*Those marked * are Members of the Society.*

| | |
|---|---|
| Ackland, J. McKno ... ... ... ... ... ... | 1 Barnfield Crescent, Exeter. |
| *Ackland, W. R. ... ... ... ... ... ... ... | 5 Rodney Place, Clifton. |
| Adye, W. J. A. ... ... ... ... ... ... ... .. | Church House, Bradford, Wilts. |
| Aldous, George F. ... ... ... ... ... ... | Charlton House, Mannamead, Plymouth. |
| *Alexander, D.A., M.B., Ch.B. Vict.... ... ... | 30 Berkeley Square, Clifton. |
| *Almond, G. H. H., M.A., M.B., B.Ch. Oxon. | Lynvale, St. Mark's Place, Bath. |
| *Ambrose, J., M.B. C.M. Aberd. ... ... ... ... | 138 Fishponds Road, Eastville, Bristol. |
| *Aubrey, T., M.B. Lond. ... ... ... ... ... | Bitton, near Bristol. |
| Aveline, H. T. S. ... ... ... ... ... ... ... | Somerset Asylum, Taunton. |

| | |
|---|---|
| *Ballance, H. Stanley M.D. Lond. ... ... | 4 Royal Terrace, Weston-super-Mare. |
| Barber, M. C. ... ... ... ... ... ... ... | Staple Hill, Bristol. |
| Baretti, T. G. L'Enard ... ... ... ... ... | 49 Royal York Crescent, Clifton. |
| *Barker, G. H., M.D., B.Sc. Lond. ... ... ... | 124 Redland Road, Bristol |
| *Baron, Barclay J., M.B., C.M. Ed.... ... ... | 16 Whiteladies Road, Clifton. |
| Basset, Walter ... ... ... ... ... ... ... | 24 Stow Hill, Newport, Mon |
| Batten, W. Smith... ... ... ... ... ... ... | West Hill, Calne. |

### LIST OF SUBSCRIBERS.

| | |
|---|---|
| Bayer Co., The | 19 St. Dunstan's Hill, London, E.C. |
| *Benson, J. R. | 11 The Circus, Bath. |
| *Bergin, F. G. | 12 West Park, Clifton. |
| Bernard, C. | 2 Spencer Terrace Fishponds. |
| *Berry, F. C., M.D., B.Ch., B.A. Dub. | Burnham, Somerset. |
| Berry, James, M.B., B.S. Lond. | 21 Wimpole Street, London, W. |
| Biamonti, Sebastiano | 27 Via Balbi, Genoa, Italy. |
| *Blacker, A. E. | 20 Victoria Square, Clifton. |
| Blackett, J. F., M.D., B.S. Lond. | Audley, Newcastle, Staffs. |
| *Blackley, F. J., M.D. Dub. | 2 Knowle Road, Bristol. |
| *Blagg, Arthur F., M.D. Durh. | 28 Caledonia Place, Clifton. |
| Bodman, Hervey, M.D. Lond. | 9 Whiteladies Road, Clifton. |
| *Boucher, F. T. | 31 Downleaze, Stoke Bishop. |
| *Bowker, G. E., M.D. Edin. | 11 North Parade, Bath. |
| Boyce, James G. | 17 Melrose Place, Clifton. |
| Boyd, Geo. S. J. | 19 St. George's Square, Stamford, Lincs. |
| *Brasher, C. W. J., M.B., Ch.B. Bristol | 128 Cotham Brow, Bristol. |
| *Brew, R. H. | Chew Magna. |
| *Bristowe, H. C., M.D. Lond. | Wrington, Somerset. |
| *Brown, William, M.D., C.M. Glasg. | Park View, Fishponds, Gloucestershire. |
| *Bullen, F. St. John | 3 Richmond Park Road, Clifton. |
| Burroughs, E. F. H. | 27 Burlington Road, Redland, Bristol. |
| *Bush, J. Paul, C.M.G. | Vyvyan House, Clifton Park, Clifton. |

| | |
|---|---|
| *Cadman, E. S. R., M.D., C.M. Edin. | 22 Trelawney Road, Cotham. |
| *Carling, A., M.A., M.B., B.C. Cantab. | 12 Great George Street, Bristol. |
| *Carter, T. M., M.D. Durh. | Burnley, Westbury-on-Trym. |
| *Carwardine, Thomas, M.B., M.S. Lond. | 16 Victoria Square, Clifton. |
| *Cave, Edward J., M.D. Lond. | 20 Circus, Bath. |
| *Charles, J. R., M.A., M.D., B.C. Cantab. | 12 Victoria Square, Clifton. |
| *Chitty, H., M.S. Lond. | 46 Pembroke Road, Clifton. |
| Chute, Henry M. | King William's Town, S. Africa. |
| *Clarke, C., M.B., B.S. Lond. | Brompton Hospital, London. |
| *Clarke, John Michell, M.D., M.A. Cantab. | 28 Pembroke Road, Clifton. |
| *Clarke, R. C., M.B., Ch.B. Bristol | Royal Infirmary, Bristol. |
| Cobbledick, A. S., M.D., B.S. Lond. | 402 Brixton Road, London, S.W. |
| Coleridge, Alfred, M.B., B.S. Lond. | Cross Tree House, Moretonhampstead, Devon. |
| Connellan, Lieut. P. S., I.M.S. | c/o Grindlay & Co., 54 Parliament Street, London. |
| *Cook, Henri, M.D., C.M. Aberd. | 1 Dean Lane, Southville, Bristol. |
| *Coombs, Carey, M.D. Lond. | 3 Pembroke Road, Clifton. |
| *Corfield, C. | 12 Pembroke Road, Clifton. |
| Cossham, W. R., M.D., C.M. Aberd. | Cirencester. |
| Cotton, William, M.D., C.M., M.A. Ed. | 231 Gloucester Road, Bristol. |
| Coulter, R. J., M.B. Dub. | 11 Clytha Park Road, Newport, Mon. |
| Cridland, A. B. | 52 Waterloo Road, Wolverhampton. |
| *Cross, F. Richardson, M.B. Lond. | Worcester House, Clifton. |
| *Crossman, F. W. | Hambrook, Gloucestershire. |
| *Crouch, C. Percival, M.B. Lond. | 1 Royal Terrace, Weston-super-Mare. |

| | |
|---|---|
| *Dacre, John | 14 Eaton Crescent, Clifton. |
| *Davies, D. S., M.D. Lond., D P.H. Cantab. | 60 Oakfield Road, Clifton. |

### LIST OF SUBSCRIBERS.

| | |
|---|---|
| Dening, Edwin | Manor House, Stow-on-the-Wold. |
| Devine, H., M.D., Lond. | West Riding Asylum, Wakefield. |
| Dickie, James Steel, M.D., C.M. Aberd. | Boscombe Park, Bournemouth. |
| *Dickinson, W. G., M.D., Durh. | Wood Hill, Adelaide Terr., Portishead. |
| *Dixon, T. B. | 91 Coronation Road, Bristol. |
| *Donaldson, R., M.A., M.B., Ch.B. Edin. | 20 Abbotsford Road, Cotham. |
| *Dowling, E. A. G. | 5 Rodney Place, Clifton. |
| Dunbar, Eliza L. Walker, M.D. Zürich | 9 Oakfield Road, Clifton. |
| Dymoke, F. | 21 Cotham Road, Bristol. |

| | |
|---|---|
| Eadon, W. F. Bailey | Hambrook, Gloucestershire. |
| *Eager, Reginald, M.D. Lond. | Northwoods, Winterbourne. |
| *Edgeworth, F. H., M.D., B.C., B.A. Cantab., D.Sc. Lond. | 4 Richmond Park Road, Clifton. |
| Edwards, E. S. | Empingham, Stamford. |
| Elder, William | 4 Trelawney Road, Cotham. |
| Elliot Bros. Ltd. | *Australian Pharmaceutical Notes & News*, O'Connell Street, Sydney, N.S.W. |
| *Elliott, Christopher, M.D., B.A. Dub. | 102 Pembroke Road, Clifton. |
| Elliott, F. Percy, M.B., C.M. Aberd. | 113 Grove Road, Walthamstow, London. N.E. |
| *Elliott, W. H. A., M.B., B.S. Lond. | Rockfort, Arley Hill, Bristol. |
| *Every-Clayton, L. E. V., M.D. Lond. | Rosslyn, Clevedon, Somerset. |

| | |
|---|---|
| *Farquhar, J., M.D. Aberd. | 11 Dublin Crescent, Henleaze Road, Bristol. |
| *Fells, A., M.B., C.M. Edin. | 107 Ashley Road, Bristol. |
| *Ferguson, R. S., B.A. Durh., M.B. C.M. Edin. | Elm Grove, Calne, Wilts. |
| *Firth, J. L., M.D., M.S. Lond. | 8 Victoria Square, Clifton. |
| *Fisher, A. G. T. | St. Francis Vicarage, Ashton Gate. |
| *Flemming, A. L., M.B., Ch.B. Bristol | 34 Alma Road, Clifton. |
| *Flemming, C. E. S. | Manvers House, Bradford-on-Avon. |
| *Fortescue-Brickdale, J. M., M.A., M.D., B.Ch. Oxon. | 52 Pembroke Road, Clifton. |
| Foss, E. V., M.B., Ch.B. Bristol | Clouds Hill House, St. George, Bristol. |
| Fox, F. F. | Yate House, Yate. |
| *Fraser, Forbes | 2 The Circus, Bath. |
| Freeman, J., M.D. Brux. | Waterloo Villa, Queen's Road Clifton. |

| | |
|---|---|
| Garland, E. B., M.B., C.M. Edin. | 114a Hampton Road, Redland. |
| *Gee, C. A. H., M.B., Ch.B. Bristol | Evercreech, Somerset. |
| Genge, T. T. | 21 Whiteladies Road, Bristol. |
| *Gerrish, D. S. | 322 Church Road, St. George, Bristol. |
| *Gibbs, A. N. Godby | 52 Whiteladies Road, Clifton. |

### LIST OF SUBSCRIBERS.

| | |
|---|---|
| *Gratte, C. Brooke | 19 Gold Tops, Newport, Mon. |
| *Green, T. A., M.D., C.M. Edin. | 33a Whiteladies Road, Clifton. |
| Green-Armytage, Capt. V. B., I.M.S. | c/o King & Co., Bombay. |
| *Greer, W. J. | 19 Gold Tops, Newport, Mon. |
| *Grey, Harry, M.D., C.M.Edin. | 492 Fishponds Road, Bristol. |
| Griffiths, Cornelius A. | 6 Windsor Place, Cardiff. |
| *Griffiths, J. S. | 20 Redland Park, Bristol. |
| *Groves, E. W. H., M.D., M.S. Lond. | 16 Richmond Hill, Clifton. |

| | |
|---|---|
| *Hailes, Clements D. G., M.D., C.M. Ed. | 27 Alma Road, Clifton. |
| *Hall, E. G., M.B. Lond. | 53 Hampton Road, Redland, Bristol. |
| *Hall, I. Walker, M.D., Ch.B.Vict. | Linden Gate, Clifton Down Road, Clifton. |
| Handfield-Jones, Montagu, M.D. Lond. | 35 Cavendish Square, London, W. |
| *Harris, H. Elwin, B.A., M.B. Cantab. | 13 Lansdown Place, Clifton. |
| *Harsant, W. H. | Tower House, Clifton. |
| Hartnell, E. B. | 13 Church Street, Bridgwater. |
| *Hawes, C. S. | 27 Clarence Parade, Southsea. |
| *Hawes, I. H., M.B., B.S. Durh. | Wick, Glos. |
| Hayman, Alfred G. | Hapsford House, near Frome. |
| *Hayman, C. A., M.D. St. And. | Kingston Villa, Richmond Hill, Clifton. |
| Hele, T. S., M.A., M.B. | Emmanuel College, Cambridge. |
| *Herapath, C. E. K., M.D. Lond. | 12 Brunswick Square, Bristol. |
| *Herapath, C. K. C. | Cotham Park, Bristol. |
| Hill, Hedley, M.D. Durh. | 1 Whiteladies Road, Clifton. |
| *Hill, Walter J. | The Brow, Clevedon. |

Hospital, Bristol General (*Sec.*, W. Thwaites).
Hospital, Taunton and Somerset (*House-Surgeon*, W. Drake).

| | |
|---|---|
| *Iles, A. E., M.B., B.S. Lond. | Cotham Lawn, Cotham Park, Bristol. |
| *Imlay, G. A., M.B., C.M. Aberd. | 1 Cotham Park, Bristol. |

Infirmary, Bristol Royal (*Secretary*, W. E. Budgett).

| | |
|---|---|
| Ingle, C. Durban | Somerton, Somerset. |
| *Irwin, S., M.B., B.Ch., B.A.O., R.U.I. | Olveston, Glos. |

| | |
|---|---|
| *James, C. W. W. | 246 Wells Road, Knowle. |
| Jamison, A., M.B., B.Ch., M.A. R.U.I. | Ardenvohr, Woodstock Road, Belfast. |
| *Joll, C.A., M.B., M.S. Lond. | 14 Elton Terrace, Bishopston. |
| *Jones, E. J. Trevor, M.D. Brux. | Aberdare, Glamorganshire. |

### LIST OF SUBSCRIBERS.

| | |
|---|---|
| *Jones, J. Ellington ... ... ... ... .. ... | 16 Richmond Park Road, Clifton. |
| Jones, H. Macnaughton, M.D., M.Ch. R.U.I. | 131 Harley Street, London, W. |
| Joseph, A. Hill, M.D. Lond. ... ... ... ... | Bexhill-on-Sea. |
| *Kemm, F. St. J.... ... ... ... ... ... ... ... | Worle. |
| *Kingston, S. H., M.B., Ch.B. Bristol ... ... | Royal Infirmary, Bristol. |
| *Kyle, H. G., M.B., B.Ch. Oxon. ... ... ... | 31 Westbury Road, Westbury-on-Trym. |
| *Lace, F. ... ... ... ... ... ... ... ... ... ... | 5 Gay Street, Bath. |
| *Lansdown, R. G. P., M.B., B.S. Dur. ... ... | 39 Oakfield Road, Clifton. |
| *Lavington, C. C., M.B., B.S. Dur. ... ... | 1 Burlington Road, Redland. |
| Lendon, A. A., M.D. Lond. ... ... ... ... | North Terrace, Adelaide, S. Australia. |
| Le Soudier, H. ... ... ... ... ... ... ... ... | 174 et 176 Boulevard St. Germain, Paris |
| *Lees, E. Leonard, M.D., C.M. Ed. ... ... ... | 1 Chesterfield Place, Clifton. |
| Leigh, W. W. ... ... ... ... ... ... ... ... | Treharris, Glamorganshire. |
| *Leonard, R. C., M.D. Ed. ... ... ... ... ... | 134 Coronation Road, Bristol. |
| *Llewellyn, R. Ll. J., M.B. Lond. ... ... ... | 41 Gay Street, Bath. |
| *Lucas, J. J. S., M.D. Lond. ... ... ... ... ... | 186 Wells Road, Totterdown. |
| Lucy, Reginald H. ... ... ... ... ... ... | 9 The Crescent, Plymouth. |
| *McCrea, P. W., M.D., B.Ch., B.A.O., B.A. Dub. ... ... ... ... ... ... ... ... | 19 Portland Square, Bristol. |
| Mac Donald, P. W., M.D., C.M. Aberd.... ... | Herrison, Dorchester. |
| Mackinnon, Charles, M.B., C.M., M.A. Glasg. | Davaar, Cirencester. |
| Maclean, Ewen J., M.D., C.M. Ed.... ... ... | 12 Park Place, Cardiff. |
| Maclean, J. Campbell, M.B., C.M. Ed.... ... | Swindon House, Swindon, Wiltshire. |
| *Macleod, D., M.B., Ch.B. Ed. ... ... ... ... | 84 Redland Road, Bristol. |
| *Macphail, J. M., M.B., Ch.B. Ed. ... ... ... | Eastern Dispensary, Bath. |
| *MacWatters, J. C. .. ... ... ... ... ... | Almondsbury. |
| Marsh, O. E. Bulwer ... ... ... ... ... ... | Parkdale, Newport, Mon. |
| Marshall, Arthur L., M.B., B.A. Cantab ... | 206 Upper Clapton Road, London, N.E. |
| *Martin, E. F., M.B. Ed. ... ... ... ... ... | 7 Royal Terrace, Weston-super-Mare. |
| *Martin, R. C. ... ... ... ... ... ... ... ... | 3 Clifton Vale, Clifton. |
| *Martyn, G. J. King, M.D., B.C., B.A. Cantab. ... ... ... ... ... ... ... ... ... | 12 Gay Street, Bath. |
| Mason, S. B. ... ... ... ... ... ... ... ... | 12 Park Terrace, Pontypool. |
| Mason, William ... ... ... ... ... ... ... | Sedgemoor Cottage, St. Austell, Cornwall. |
| Miall, C. L. ... ... ... ... ... ... ... ... | Elm Hayes, Paulton, near Bristol. |
| *Mole, Harold F. ... ... ... ... ... ... ... | 19 Mortimer Road, Clifton, Bristol. |

### LIST OF SUBSCRIBERS.

*Moore, C. A., M.B., M.S. Lond. ... ... ... ... General Hospital, Bristol.

*Moore, L. A., M.B., Ch.B., Bristol ... ... ... 45 Ashley Road, Bristol.

*Morton, C. A. ... ... ... ... ... ... ... ... 14 Vyvyan Terrace, Clifton.

*Mumford, W. G., M.B. Lond. ... ... ... ... 18 The Circus, Bath.

Munden, Charles ... ... ... ... ... ... ... ... Ilminster.

*Munro, J. M. H., D.Sc. Lond. ... ... ... ... 12 Grosvenor Place, Bath.

*Myles, G T. ... ... ... ... ... ... ... ... 7 Apsley Road, Clifton.

*Neild, Newman, M.B., B.Ch. Vict. ... ... ... 9 Richmond Hill, Clifton.
*Newman, Herbert, M.B., B.S.Dur. ... ... 150 Wells Road, Bristol.
*Newnham, W. H. C., M.B., M.A. Cantab. ... 3 Lansdown Place, Victoria Square, Clifton.
*Nichols, F. C., M.B., Ch.B. Bristol ... ... ... 38 Whiteladies Road, Clifton.
*Nixon, J. A., B.A., M.B., B.C. Cantab. ... ... 19 Mortimer Road, Clifton.
Norman, J. H. ... ... ... ... ... ... ... ... Goodleigh, Winkleigh, Devonshire.

Odell, William, M.D. Durh. ... ... ... ... Ferndale, Torquay.
*Ogilvy, Alexander, M.D., B.Ch., B.A. Dub. Leny, Clifton Park, Clifton.
Openshaw, E. H. ... ... ... ... ... ... ... ... Cheddar.
*Opie, P. A., B.C. Cantab. ... ... ... ... ... Royal Infirmary, Bristol.
Oppenheimer, Son & Co. Ltd. ... ... .. 179 Queen Victoria Street, E.C.
*Ormerod, H. Lawrence, M.B., B.Ch. R.U.I. 2 Henleaze Road, Westbury Park.

Page, G. Shepley ... ... ... ... ... ... ... ... 78 Old Market Street, Bristol.

*Parker, George, M.D., M.A. Cantab. ... ... 14 Pembroke Road, Clifton.

Parkinson, Lt. G. S., R.A.M.C. ... ... ... c/o Messrs. Holt & Co., Whitehall, S.W.

*Parsons, H. F.... ... ... ... ... ... ... ... The Heath, Stoke Bishop.

Parsons, J. H., M.B., B.S., D.Sc. Lond.... ... 54 Queen Anne Street, London, W.

Paterson, Donald Rose, M.D., C.M. Ed. ... 18 Windsor Place, Cardiff.

Peake, G. Arthur ... ... ... ... ... ... ... Rodney Place, Cheltenham.

*Pearse, J., M.D., C.M. Ed.... ... ... ... ... 28 St. George's Terrace, Trowbridge.

Phillips, C. M., M.D. Brux. ... ... ... ... Ravenslea, Kensington Hill, Brislington

*Pickering, C. F. ... ... ... ... ... ... ... ... 13 Berkeley Square, Bristol.

*Pinniger, W. J. H., M.D., B.S. Lond. ... ... 75 Effingham Road, Bristol.

*Pineo, E. G. D.... ... ... ... ... ... ... ... Richmond House, Langford, nr. Bristol.

*Porteous, H. B., M.B., Ch.B. Ed. ... ... ... Newbury House, Weston-super-Mare.

Powell, J. J., M.D. Lond. ... ... ... ... ... 2 Gloucester Road, Bishopston, Bristol.

Powell, William, M.B. Lond. ... ... ... ... Hill Garden, Torquay.

Price, D. T. M.B. Lond. ... ... ... ... ... Castle Cary, Somerset.

### LIST OF SUBSCRIBERS.

*Prichard, Arthur W. ... ... ... ... ... ... ... 6 Rodney Place, Clifton.
*Prowse, Arthur B., M.D. Lond. ... ... ... 5 Lansdown Place, Clifton.
Prowse, W. B. ... ... ... ... ... ... ... ... 31 Vernon Terrace, Brighton.

Randolph, Charles ... ... ... ... ... ... St. Michael's, Milverton, Somerset.
*Rattray, J. M., M.D., C.M., M.A. Aberd. ... Frome.
*Rayner, D. C. ... ... ... ... ... ... ... ... 9 Lansdown Place, Clifton, Bristol.
Rendall, John ... ... ... ... ... ... ... Forestside, Lymington.
Ritchie, Thomas P. ... ... ... ... ... ... 27 Oakfield Road, Clifton.
*Robertson, D., M.B., Ch.B. Edin. ... ... ... 188 Wells Road, Knowle.
*Rogers, B. M. H., M.D., B.Ch., B.A. Oxon. ... 1 Victoria Square, Clifton.
*Rolfe, R., B.A., M.B., B.C. Cantab. ... ... Park House, Avonmouth.
*Rose, F. H. ... ... ... ... ... ... ... ... ... 13 Westfield Park, Clifton.
*Rossiter, George F., M.B. Lond. ... ... Cairo Lodge, Weston-super-Mare.
*Roxburgh, Robert, M.B. Ed. ... ... ... 1 Victoria Buildings, Weston-super-Mare.
*Rudge, C. King ... ... ... ... ... ... ... ... 145 Whiteladies Road, Bristol.
*Rutherford, J. M., M.B., C.M. Edin. ... ... Brislington House, Bristol.

*Savage, W. G., M.D. Lond., D.P.H. ... ... Hafton, Uphill Road, Weston-s.-Mare.
*Schölberg, H. A., M.B., B.S. Lond. ... ... Greenbank, Llanishen, Glam.
Scott, Baty- ... ... ... ... ... ... ... ... 28 Belluton Road, Knowle, Bristol.
Seward, W. J., M.B. Lond. ... ... ... ... Colney Hatch Asylum, New Southgate, London, N.
Sheppard, Lieut. A. L., I.M.S., M.B., B.S. Dur. c/o Grindlay, Groom & Co., Bombay.
*Short, A. R., M.D., B.S., B.Sc. Lond. ... ... 2 Westbourne Place, Clifton.
*Short, L. J., M.D., B.Sc. Lond. ... ... ... Tuberculosis Dispensary, Woolwich, S.E.
Skelton, Henry, M.D. St. And. ... ... ... Downend, Gloucestershire.
Smith, Capt. F. Shingleton, I.M.S. ... ... c/o Grindlay & Co., Bombay.
*Smith, G. Cockburn, M.D. Brux. ... ... ... 14 South Road, Newton Abbot.
*Smith, G. Munro ... ... ... ... ... ... ... ... 18 Apsley Road, Clifton.
Smith, L. Shingleton, B.A., M.B. Cantab.... Honddu House, Brecon.
Smith, Rev. Reginald, M.A. Cantab. ... ... The Vicarage, Walpole St. Andrew, Norfolk.
Smith, W. Steele, M.B. Lond. ... ... ... 1 Wilbraham Rd., Chorlton-cum-Hardy, Manchester.
*Smith, W. Alexander, M.B., M.A. Oxon. ... 70 Pembroke Road, Clifton.
Society, Cardiff Medical (*Hon. Lib.*, Queen Street, Cardiff).

| | |
|---|---|
| SOCIETY, MANCHESTER MEDICAL ... ... ... | Medical Society's Library, The Owens College, Manchester. |
| SOCIETY, PLYMOUTH MEDICAL (*Hon. Lib.*, J. ELLIOTT SQUARE). | |
| *STACK, E. H. E., B.A., M.B., B.C. Cantab. ... | Arvalee, Clifton Down Road, Clifton. |
| STAPLE, J. D. ... ... ... ... ... ... ... ... | 153 Cheltenham Road, Bristol. |
| STATHAM, R. WHITESIDE ... ... ... ... ... | Cheddar, Somersetshire. |
| *STEELE, CHARLES, M.D. Durh. ... ... ... ... | 17 Richmond Hill, Clifton. |
| STEVENS, W. H. ... ... ... ... ... ... ... | 26 Zetland Road, Bristol. |
| *STOCK, W. S. V., M.B. B.S Lond. ... ... ... | Montrose House, Queen's Road, Clifton |
| STODDART, F. WALLIS ... ... ... ... ... ... | Southey House, College Green, Bristol. |
| *SWAIN, JAMES, M.D., M.S. Lond. ... ... ... | 4 Victoria Square, Clifton. |
| *SWAYNE, W. CARLESS, M.D. Lond. ... ... ... | 56 St. Paul's Road, Clifton. |
| *SYMES, J. O., M.D. Lond. ... ... ... ... ... | 71 Pembroke Road, Clifton. |
| SYMONDS, H. P. ... ... ... ... ... ... ... | 35 Beaumont Street, Oxford. |
| TAYLER, H. P., M.B., B.C. Cantab. ... ... ... | The Abbey House, Bradford-on-Avon. |
| TAYLOR, A. L. ... ... ... ... ... ... ... ... | 22 Downfield Road, Clifton. |
| TAYLOR, C. J. ... ... ... ... ... ... ... ... | 191 Luton Road Chatham. |
| *TAYLOR, JAMES ... ... ... ... ... ... ... ... | 36 Alma Road, Clifton. |
| *TAYLOR, J. W., M.B., Ch.B. Bristol ... ... ... | 8 West Redcliff Parade, Bristol. |
| *TEMPLE, G. H., M.B., C.M. Ed. ... ... ... | Ailanthus, Weston-super-Mare. |
| THIN, JAMES ... ... ... ... ... ... ... ... | 54 & 55 South Bridge, Edinburgh. |
| THOMAS, A. GARROD, M.D., C.M. Ed. ... ... | Clytha Park, Newport, Mon. |
| *THOMAS, J. D., M.B. Cantab. ... ... ... ... | Northwoods, Winterborne, |
| THOMAS, J. LYNN, C.B. ... ... ... ... ... ... | 21 Windsor Place, Cardiff. |
| *THOMAS, J. M. M. ... ... ... ... ... ... ... | 5 Colston Parade, Redcliff, Bristol. |
| *THOMAS, J. TUBB, D.P.H. ... ... ... ... ... | The Halve House, Trowbridge. |
| *THOMAS, R. E., M.D., B.S. Lond. ... ... ... | The Mount, Newport, Mon. |
| THOMSON, EUSTACE B., M.D., C.M. Glasg. ... | Albany Place, Plymouth. |
| *THOMSON, F. G., M.A., M.D. Lond. ... ... ... | 20 Gay Street, Bath. |
| TOSSWILL, LEONARD R., D.P.H., Cantab. ... | Devon County Council Offices, 14 Bedford Circus, Exeter. |
| VACHELL, HERBERT R., M.D., C.M. Aberd. ... | 18 Newport Road, Cardiff. |
| *VEALE, P. J., M.B., B.S. Lond. ... ... ... ... | 17 Oakfield Road, Clifton. |
| *VICKERY, W. H. ... ... ... ... ... ... ... | 1 Trewartha Park, Weston-super-Mare. |
| *WALDO, HENRY, M.D., C.M. Aberd. ... ... ... | 19 Pembroke Road, Clifton. |
| *WALKER, CYRIL H., M.B., B.A. Cantab. ... ... | 8 Oakfield Road, Clifton. |
| WALLACE, REV. CANON, M.A. Oxon. ... ... ... | 3 Gloucester Row, Clifton. |
| *WALLACE, JOHN ... ... ... ... ... ... ... | Lauriston, 15 Knightstone Road, Weston-super-Mare. |
| WALLACE, J. T., M.B., B.Ch., B.A. R.U.I. ... | 48 Coronation Road, Bedminster. |
| *WALLACE, JAMES W. ... ... ... ... ... ... | 1 Greville Road, Southville, Bristol. |
| *WALLIS, G. F. C., M.B., Ch.B. Ed. ... ... ... | The Cedars, Aldershot. |
| *WALTERS, C. F. ... ... ... ... ... ... ... | 19 Whatley Road, Clifton. |
| *WATERHOUSE, R., M.D. Lond. ... ... ... ... | 28 Gay Street, Bath. |
| *WATSON-WILLIAMS, P., M.D. Lond. ... ... ... | 2 Rodney Place, Clifton. |
| WEBBER, WILLIAM W. ... ... ... ... ... ... | Crewkerne. |

| | |
|---|---|
| *WHICHER, A. H. | 115 Queen's Road, Clifton. |
| WHITE, C. R. | Nixon Villa, Merthyr Vale, Glamorgan. |
| WHITE, J. W. | Orchard House, Nailsea. |
| *WHITE, PERCY W. | College Green, Bristol. |
| WIGMORE, J., M.D. R. U. I. | 16 Queen Square, Bath. |
| WILCOX, HENRY, M.B. Aberd. | 10 Willington Road, Eastbourne. |
| WILLCOX, R. LEWIS | Warminster. |
| WILLETT, GEORGE G. D. | Keynsham, Somerset. |
| *WILLIAMS, E. C., M.B., B.C., B.A. Cantab. | 159 Whiteladies Road, Clifton. |
| WILLIAMS, H. EGERTON | The Mount, Newport, Mon. |
| WILLIAMS, S. R., M.B. Lond. | Buckfastleigh, Devon. |
| *WILLIAMS, W. ROGER | Morven, Walton-by-Clevedon. |
| *WILLIAMSON, G. SCOTT | Bristol General Hospital. |
| WILLIS, W. M. | 7 Regent Street, Nottingham. |
| *WILLS, W. K., M.B., B.C. Cantab. | 19 Whiteladies Road, Clifton. |
| *WINDSOR-AUBREY, H. W. | Coronation Road, Bristol. |
| *WOOD, W. V. | Langford, near Bristol. |
| WOOLLCOMBE, WALTER L. | 16 The Crescent, Plymouth. |
| *WRIGHT, A. J., M.B., B.S. Lond. | 60 St. Paul's Road, Clifton. |
| YOUNG, J., M.D. Glas. | 14 Chantry Road, Clifton. |

THE TRANSACTIONS

OF THE

OPHTHALMOLOGICAL SOCIETY

OF THE UNITED KINGDOM

*is now issued in two forms:*

(*a*) AS FASCICULI (THREE ANNUALLY AT FOUR SHILLINGS EACH),

(*b*) BOUND IN A VOLUME COMPLETE, AS HERETOFORE (12/6 net)

*The Sale of the Transactions is not limited to Members of the Society. They may be purchased, in either form, of the Publishers, to whom also application for back volumes should be made.*

J. & A. CHURCHILL, 7 GREAT MARLBOROUGH STREET, LONDON.

### Bristol Medico-Chirurgical Journal,

MARCH, 1912.

### EXCHANGE-LIST.

### AFRICA.

#### CAPE COLONY.

*Monthly.*

*South African Medical Record.* Cape Town: Townshend, Taylor & Snashall.

### AMERICA.

#### ARGENTINE REPUBLIC.

*Weekly.*

*La Semana Médica.* Buenos Ayres: 737 Callao.

#### CANADA

*Monthly.*

*The Canada Lancet.* Toronto: The Ontario Publishing Co. Ltd.

*The Canadian Medical Association Journal.* Toronto: Morang & Co. Ltd.

#### MEXICO.

*Twice a month.*

*Gaceta Médica.* Mexico: Apartado postal, 517.

#### PERU.

*Twice a month.*

*La Crónica Médica.* Lima: Apartado postal, No. 629.

#### UNITED STATES.

*Weekly.*

*Boston Medical and Surgical Journal.* Boston: 101 Tremont Street.

*Medical Record.* New York: William Wood & Co.

*The Lancet-Clinic.* Cincinnati: Miami Building.

*Monthly.*

*American Journal of Surgery.* New York: 92 William Street.

*American Medicine.* Burlington: 189 College Street.

*Archives of Pediatrics.* New York. E. B. Treat & Co.

*Buffalo Medical Journal.* Buffalo: 238 Delaware Avenue.

*Bulletin of the Johns Hopkins Hospital.* Baltimore: The Johns Hopkins Press.

*California State Journal of Medicine.* San Francisco: Butler Building.

*New York State Journal of Medicine.* New York: 17 West 43rd Street.

*St. Louis Medical Review.* St. Louis: Metropolitan Building.

*The American Journal of Obstetrics.* New York: William Wood & Co.

*The American Journal of Ophthalmology.* St. Louis: 316 Metropolitan Building.

*The Monthly Cyclopædia of Practical Medicine.* Philadelphia: The F. A. Davis Co.

*The Post-Graduate.* New York: 2nd Avenue and 20th Street.

*The Therapeutic Gazette.* Detroit: E. G. Swift.

*Quarterly.*

*The Alienist and Neurologist.* St. Louis: 385 West Pine Street.

*Irregularly.*

*The Journal of Experimental Medicine.* Lancaster, Pa.: The New Era Printing Co.

*The Journal of Medical Research.* Boston: 240 Longwood Avenue.

*Yearly.*

*Smithsonian Report.* Washington: Government Printing Office.

*Studies from the Department of Pathology of the College of Physicians and Surgeons—Columbia University,* New York: 437 West 59th Street.

*Transactions of the American Association of Obstetricians and Gynecologists.* Philadelphia: William J. Dornan.

*Transactions of the American Dermatological Association.* New York: The Grafton Press.

*Transactions of the American Laryngological Association.* New York: The McConnell Printing Co.

*Transactions of the American Ophthalmological Society.* Hartford: The Case, Lockwood & Brainard Co.

*Transactions of the American Pediatric Society.* New York: E. B. Treat & Co.

*Transactions of the American Surgical Association.* Philadelphia: William J. Dornan.

*Transactions of the Association of American Physicians.* Philadelphia: William J. Dornan.

*Transactions of the College of Physicians of Philadelphia.* Philadelphia: William J. Dornan.

### ASIA

#### CHINA.

*Quarterly*

*The China Medical Missionary Journal.* Shanghai: 18 Peking Road.

#### INDIA.

*Monthly.*

*The Antiseptic.* Madras: U. Rama Rau.

*The Indian Medical Gazette.* Calcutta: Thacker, Spink & Co.

*Practical Medicine* Delhi: The Medical Scientific Press.

#### JAPAN.

*Monthly.*

*The Sei-I-Kwai Medical Journal.* Tokyo: Charity Hospital Medical College.

#### PHILIPPINE ISLANDS.

*Bi-monthly.*

*The Philippine Journal of Science* Manila: Bureau of Printing.

### AUSTRALASIA.

### AUSTRALIA.

*Weekly.*

*The Australian Medical Journal.* Melbourne: Charles J. Steel.

### NEW ZEALAND.

*Quarterly.*

*New Zealand Medical Journal.* Wellington: B.M.A. (Wellington Branch).

### EUROPE

### AUSTRIA.

*Weekly.*

*Wiener klinische Wochenschrift.* Vienna: Wilhelm Braumüller.

### BELGIUM.

*Monthly.*

*Bulletin de l'Académie royale de Médecine de Belgique* Brussels: F. Hayez.

### BRITISH ISLES.

*Weekly.*

*British Medical Journal.* London: 429 Strand.

*Medical Press and Circular.* London: A. A. Tindall.

*The Clinical Journal.* London: The Medical Publishing Company, Limited.

*The Hospital.* London: The Scientific Press (Limited).

*The Lancet.* London: 423 Strand.

*The Sanitary Record.* London: The Sanitary Publishing Co., Ltd.

*Fortnightly.*

*The Journal of Tropical Medicine.* London: John Bale, Sons & Danielsson, Ltd.

*Monthly.*

*Edinburgh Medical Journal.* Edinburgh: Wm. Green & Sons.

*Public Health.* London: The Society of Medical Officers of Health.

*The Birmingham Medical Review.* Birmingham Percival Jones, Ltd.

*The British Journal of Dermatology.* London: H. K. Lewis.

*The Glasgow Medical Journal.* Glasgow: Alex. Macdougall.

*The Journal of Laryngology, Rhinology, and Otology,* London: Adlard & Son.

*The Medical Review.* London: 66 Finsbury Pavement.

*The Medical Chronicle.* Manchester: Sherratt & Hughes.

*The Polyclinic.* London: John Bale, Sons & Danielsson, Ltd.

*The Practitioner.* London: Howard House, Strand.

*The Prescriber.* Edinburgh: 137 George Street.

*The Proceedings of the Royal Society of Medicine.* London: Longmans, Green & Co.

*Quarterly.*

*Folia Therapeutica.* London: John Bale, Sons & Danielsson, Ltd.

*Journal of Mental Science.* London: J. & A. Churchill.

*The British Journal of Tuberculosis.* London: Baillière, Tindall & Cox.

*The Caledonian Medical Journal.* Glasgow: Alex. Macdougall.

*The Northumberland and Durham Medical Journal.* Newcastle-upon-Tyne: T. M. Grierson.

*The West London Medical Journal.* London: Adlard & Son.

*Half-yearly.*

*The Liverpool Medico-Chirurgical Journal.* Liverpool: Medical Institution.

*Yearly.*

*Guy's Hospital Reports.* London: J. & A. Churchill.

*King's College Hospital Reports.* London: Adlard & Son.

*Ophthalmological Transactions.* London: J. & A. Churchill.

*St. Bartholomew's Hospital Reports.* London: Smith, Elder & Co.

*St. Thomas's Hospital Reports.* London: J. & A. Churchill.

*The Medical Annual.* Bristol: John Wright & Co.

*The Medical Society's Transactions.* London: Harrison & Sons.

*The Transactions of the Edinburgh Obstetrical Society.* Edinburgh: Oliver and Boyd.

*The Westminster Hospital Reports.* London: Henry J. Glaisher.

*Transactions of the Edinburgh Medico-Chirurgical Society.* Edinburgh: James Thin.

*Transactions of the Royal Academy of Medicine in Ireland.* Dublin: John Falconer.

*Year-Book of Scientific and Learned Societies.* London: C. Griffin & Co., Ltd.

### DENMARK.

*Weekly.*

*Hospitalstidende.* Copenhagen: Jacob Lund.

### FRANCE.

*Three times a week.*

*Gazette des Hôpitaux.* Paris: 49 Rue St.-André-des-Arts.

*Weekly.*

*Bulletin de l'Académie de Médecine.* Paris: Masson et Cie

*Gazette des Hôpitaux de Toulouse.* Toulouse: 13 Rue Ste-Ursule.

FRANCE—*Weekly* (continued).

*Gazette hebdomadaire des Sciences médicales de Bordeaux.* Bordeaux: 8 Rue Paul-Bert.

*L'Echo médical du Nord.* Lille: 128 Boulevard de la Liberté.

*Le Progrès médical.* Paris: 41 Rue des Ecoles.

*Revue hebdomadaire de Laryngologie, d'Otologie et de Rhinologie.* Bordeaux: G. Gounouilhou.

*Fortnightly.*

*Gazette des Praticiens.* Lille: Rue des Guinguettes, 18.

*Twice a month.*

*Gazette de Gynécologie.* Paris: 8bis Rue de Châteaudun.

*Revue de Thérapeutique médico-chirurgicale.* Paris: Félix Alcan.

*Monthly.*

*Journal d'Hygiène.* Paris: 79 Avenue de Wagram.

*Revue générale d'Ophtalmologie.* Paris: Masson et Cie.

GERMANY.

*Weekly*

*Zentralblatt für Chirurgie.* Leipzig: J. A. Barth.

*Zentralblatt für Gynäkologie.* Leipzig: J. A. Barth.

*Zentralblatt für innere Medicin.* Leipzig: J. A. Barth.

*Münchener medicinische Wochenschrift.* Munich: J. F. Lehmann.

GREECE.

*Monthly.*

*Ιατρικὴ πρόοδος.* Syra: Renieri Brindesi.

*La Grèce médicale.* Syra: Renieri Brindesi.

*Three times a year.*

*Δελτίον τῆς ἐν Ἀθήναις Ἰατρικῆς Ἑταιρείας.* Athens: 85 rue de l' Université.

HOLLAND.

*Weekly.*

*Nederlandsch Tijdschrift voor Geneeskunde.* Amsterdam: F. van Rossen.

ITALY.

*Twice a month.*

*Giornale Internazionale delle Scienze Mediche.* Naples: Enrico Detken.

*Bi-monthly.*

*Archivio di Ortopedia.* Milan: Via San Calimero, 31.

NORWAY.

*Monthly.*

*Norsk Magazin for Lægevidenskaben.* Christiania: Steen'ske Bogtrykkeri.

RUSSIA.

*Weekly.*

*Medycyna.* Warsaw: Niecala, 7.

*St. Petersburger medicinische Wochenschrift.* St. Petersburg: K. L. Ricker.

*Monthly.*

*Finska Läkaresällskapets Handlingar.* Helsingfors: Helsingfors Centraltryckeri.

SPAIN.

*Weekly.*

*El Siglo Médico.* Madrid: Magdalena, 36.

SWEDEN.

*Bi-monthly.*

*Nordiskt Medicinskt Arkiv.* Stockholm: P. A. Norstedt & Söner.

TURKEY.

*Monthly.*

*Gazette médicale d'Orient.* Constantinople: "Levant Herald."

---

Books for Review and Exchange Journals should be sent to the Assistant-Editor, *Bristol Medico-Chirurgical Journal,* Medical Library, The University, Bristol.

### PUBLICATIONS RECEIVED.

From Edward Arnold, London:
*Practical Anatomy.* By F. G. Parsons, F.R.C.S. Eng., and William Wright, M.B., D.Sc., F.R.C.S. Eng. 2 Vols. 1912. Price, 17s. net.

From W. Austin, London:
*Report of the Proceedings of the Imperial Health Congress.* (Held under the auspices of the Women's Imperial Health Association.) 1911. Price, 1s. net.

From Baillière, Tindall and Cox, London:
*Studies in Pulmonary Tuberculosis.* By Frederick Guy Griffiths, B.A., M.D., Ch.M. 1911. Price, 5s. net.
*Medical Laboratory Methods and Tests.* By Herbert French, M.A., M.D. Third Edition. 1912. Price, 5s. net.
*The Prevention of Dental Caries and Oral Sepsis.* By H. P. Pickerill, M.D., Ch.B., M.D.S., L.D.S. 1912. Price, 7s. 6d. net.
*A Surgical Treatment of Locomotor Ataxia.* By L. N. Denslow, M.D. 1912. Price, 3s. 6d. net.
*The Treatment, Prevention and Cure of Tuberculosis and Lupus with Allyl Sulphide.* By William C. Minchin, M.D. 1912. Price, 3s. 6d. net.
*Acromegaly: A Personal Experience.* By Leonard Portal Mark, M.D. 1912. Price, 7s. 6d. net.

From John Bale, Sons & Danielsson, Ltd., London:
*On Gastroscopy.* By William Hill, B.Sc., M.D. 1912. Price, 3s. 6d. net.

From Cassell and Company, Ltd., London:
*A System of Surgery.* Edited by C. C. Choyce, M.D., F.R.C.S. Pathological Editor, J. Martin Beattie, M.A., M.D., C.M. In 3 vols. Vol. I. 1912. Price, 21s. net each volume.

From F. Davidson, London:
*Sight Testing for the General Practitioner.* By F. Davidson. Fifth Edition. 1912. Price, 2s. 6d. net.

From Henry Frowde, London:
*On Bronchial Asthma: its Pathology and Treatment.* By J. B. Berkart, M.D. Revised and abridged Third Edition. 1911. Price, 5s. net.

From J. F. Lehmann, Munich:
*Chirurgisches Vademekum für den praktischen Arzt.* Von Professor Dr. Alfred Schönwerth. 1912. Price, M. 4.

From Longmans, Green and Co., London:
*A Manual of Surgical Treatment.* By Sir W. Watson Cheyne, Bart., C.B., D.Sc., and F. F. Burghard, M.S., F.R.C.S. New Edition, by T. P. Legg, M.S., F.R.C.S., and Arthur Edmunds, M.S., F.R.C.S. Vol. I. 1912. Price, 21s. net.

From James Maclehose and Sons, Glasgow:
*A Manual of Midwifery.* By G. Balfour Marshall, M.D., C.M. 1912. Price, 14s. net.
*The Growth of Bone.* By Sir William Macewen, F.R.S. 1912. Price, 10s. net.

From Macmillan and Co., Limited, London:
*Deformities, including Diseases of the Bones and Joints.* By A. H. Tubby, M.S., F.R.C.S. Second Edition. 2 vols. 1912. Price, 45s. net.

From Alexander Mayne and Boyd, Belfast:
*Applied Anatomy of the Lungs and Pleural Membranes.* By J. Stuart Dickey, M.D., B.Ch. 1911. Price, 5s. net.

From "The Prescriber" Offices, Edinburgh:
*The Prescriber.* Vol. V. 1911.

From Smith, Elder & Co., London:
*A Junior Course of Practical Zoology.* By the late A. Milnes Marshall, M.D., D.Sc., and the late C. Herbert Hurst, Ph.D. Seventh Edition. Revised by F. W. Gamble, D.Sc., F.R.S. 1912. Price, 10s. 6d.

From John Wright & Sons, Ltd., Bristol:
*"First Aid" to the Injured and Sick.* By F. J. Warwick, M.B., and A. C. Tunstall, M.D. Seventh Edition. 1911.

